# Challenges for Prevention of Elder Abuse in Japan: Viewpoints From Legal and Practice Settings

**DOI:** 10.1093/geroni/igaa057.2376

**Published:** 2020-12-16

**Authors:** Cynthia Thomas, Tsuann Kuo

## Abstract

This symposium begins by fist comparing features of the Japanese elder abuse prevention law to 3 other abuse prevention laws - child abuse, domestic abuse and abuse against people with disabilities. It highlights amendments that have been made to the child abuse and domestic abuse prevention laws since their enactment based on implementation experience, drawing contrast to the lack of amendments made to the elder abuse prevention law despite its three-year requirement for amendment review. Overall strengths and weaknesses of the elder abuse law are discussed including needed areas of revision. Second, a case study of nearly two decades of elder abuse prevention activities in Matsudo-city are presented and the subsequent enactment in April 2020 of a new regulation of the city is discussed. In addition, interim outcomes of a related research study in Matsudo-city on elder abuse involving non-caregivers for elderly people that will expand understanding of abuse prevention efforts are presented. Third, how can we abolish elder abuse in institutional settings will be discussed by analyzing elder abuse case studies. Finally, the most updated research outcomes conducted in in-home care service settings in 2020 will be presented. Its preliminary analyses revealed out of 1,417 responses, 2.9% were found to be ”body restraints.” Such abusive actions are less likely to be detected by others. The symposium concludes by assessing challenges for preventing elder abuse in policy and practice.

